# Targeting osteoblastic casein kinase-2 interacting protein-1 to enhance Smad-dependent BMP signaling and reverse bone formation reduction in glucocorticoid-induced osteoporosis

**DOI:** 10.1038/srep41295

**Published:** 2017-01-27

**Authors:** Jin Liu, Changwei Lu, Xiaohao Wu, Zongkang Zhang, Jie Li, Baosheng Guo, Defang Li, Chao Liang, Lei Dang, Xiaohua Pan, Songlin Peng, Aiping Lu, Baoting Zhang, Ge Zhang

**Affiliations:** 1 Institute for Advancing Translational Medicine in Bone & Joint Diseases, School of Chinese Medicine, Hong Kong Baptist University, Hong Kong SAR, China; 2 Institute of Integrated Bioinfomedicine & Translational Science, School of Chinese Medicine, Hong Kong Baptist University, Hong Kong SAR, China; 3 Institute of Precision Medicine and Innovative Drug Discovery, School of Chinese Medicine, Hong Kong Baptist University, Hong Kong SAR, China; 4 Shenzhen Lab of Combinatorial Compounds and Targeted Drug Delivery, HKBU Institute of Research and Continuing Education, Shenzhen, China; 5 School of Basic Medical Sciences, Shanghai University of Traditional Chinese Medicine, Shanghai, China; 6 Department of Orthopaedics, Xi’an Third Hospital, Xi’an, China; 7 Department of Orthopaedics, Xijing Hospital, The Fourth Military Medical University, Xi’an, China; 8 School of Chinese Medicine, Faculty of Medicine, The Chinese University of Hong Kong, Hong Kong SAR, China; 9 Department of Orthopaedics and Traumatology, Bao’an Hospital Affiliated to Southern Medical University & Shenzhen 8th People Hospital, Shenzhen, China; 10 Department of Spine Surgery, Shenzhen People’s Hospital, Ji Nan University Second College of Medicine, Shenzhen, China

## Abstract

The underlying mechanism of the reduced bone formation during the development of glucocorticoid-induced osteoporosis (GIO) remains unclear. Here, we found that the highly expressed CKIP-1 together with lowly expressed total and phosphorylated Smad1/5 in bone samples was accompanied by either the reduced serum bone formation markers in GIO patients or the decreased bone formation in GIO mice. *In vitro* studies showed that the highly expressed CKIP-1 could promote Smad1 ubiquitination to suppress the Smad-dependent BMP signaling and inhibit osteogenic differentiation and mineral deposition in MC3T3-E1 cells during glucocorticoid treatment. Further, the reduced bone formation in GIO mice could not only be prevented by osteoblasts-specific Ckip-1 ablation, but also be attenuated after osteoblasts-specific Smad1 overexpression. Moreover, osteoblasts-targeting CKIP-1 siRNA treatment also attenuated the bone formation reduction in GIO mice. These study suggest that the highly expressed CKIP-1 in osteoblasts could suppress the Smad-dependent BMP signaling and contribute to the bone formation reduction in GIO. Targeting osteoblastic CKIP-1 would be a novel bone anabolic strategy for GIO patients.

Glucocorticoid-induced osteoporosis (GIO) is the most common form of secondary osteoporosis with increased fracture risk^1^,^2^. Pathologically, glucocorticoids (GC) have a detrimental effect on bone formation, turnover and integrity^3^,^4^. GIO elicits persistent reduction of bone formation, which is the major challenge in GIO treatment^1^,^2^,^3^. During the progress of GIO, the primary actions of glucocorticoids are on osteoblast lineage, wherein GC impairs the replication, differentiation and maturation as well as induce the apoptosis of osteoblasts, ultimately leading to reduced bone formation^1^,^3^. However, the molecular mechanism underlying the bone formation reduction in GIO remains largely unknown. To date, the recombinant human parathyroid hormone (iPTH) is the only bone anabolic agent clinically approved for GIO management^5^,^6^. However, iPTH treatment is limited to a 2-year period because of the increasing bone resorption over bone formation and has a potential risk of developing osteosarcoma^6^,^7^,^8^. Thus, it is desirable to investigate the molecular mechanism of bone formation reduction in GIO for developing a novel bone anabolic strategy.

The bone morphogenetic protein (BMP) signaling pathway is one of the crucial pathways responsible for osteoblastic bone formation^9^,^10^,^11^. The activation of canonical BMP signaling requires the phosphorylation of Smad1/5, which are the key signal transducers in this pathway^9^,^10^,^11^. In addition, Smad ubiquitination regulatory factor 1 (Smurf1), an E3 ubiquitin-protein ligase, acts as a major negative regulator to mediate ubiquitination and degradation of Smad1/5 in BMP signaling cascades^12^. Recently, it has been reported that casein kinase-2 interacting protein-1 (CKIP-1), a previously identified ubiquitination-related molecule, could facilitate Smurf1-mediated ubiquitination of Smad1/5 and MEKK-2 to regulate canonical Smad-dependent BMP signaling pathway and noncanonical BMP-JNK signaling pathway in HEK293T cells, respectively^13^. Although recent studies revealed that dexamethasone could significantly suppress the activation of Smad-dependent BMP signaling in osteoblastic cell lines^14^,^15^, there is no direct evidence to show the involvement of CKIP-1 in regulating the BMP signaling pathway in osteoblasts during the development of GIO.

In the present study, we found that highly expressed CKIP-1 together with lowly expressed total and phosphorylated Smad1/5 in bone samples was accompanied by either the reduced serum bone formation markers in GIO patients or the decreased bone formation rate in GIO mice, whereas we found no statistical significant difference in the intraosseous protein expression of MEKK-2 and Smurf1. Further, we performed a series of *in vitro* studies to demonstrate that highly expressed CKIP-1 could promote Smad1 ubiquitination to suppress the canonical Smad-dependent BMP signaling pathway and inhibit osteogenic differentiation and mineral deposition in MC3T3-E1 cells during GC treatment. By genetic approach, we found that the bone formation reduction in GIO mice could not only be prevented by osteoblasts-specific ablation of Ckip-1, but also be attenuated after osteoblasts-specific overexpression of Smad1. Moreover, we showed that osteoblasts-targeting CKIP-1 siRNA treatment could enhance the Smad-dependent BMP signaling and attenuate bone formation reduction in GIO mice. Together, these data suggest that targeting osteoblastic CKIP-1 could be a novel bone anabolic strategy for GIO patients.

## Results

### Highly expressed CKIP-1 associates with downregulated Smad-dependent BMP signaling and decreased bone formation in GIO

**Figure 1:**
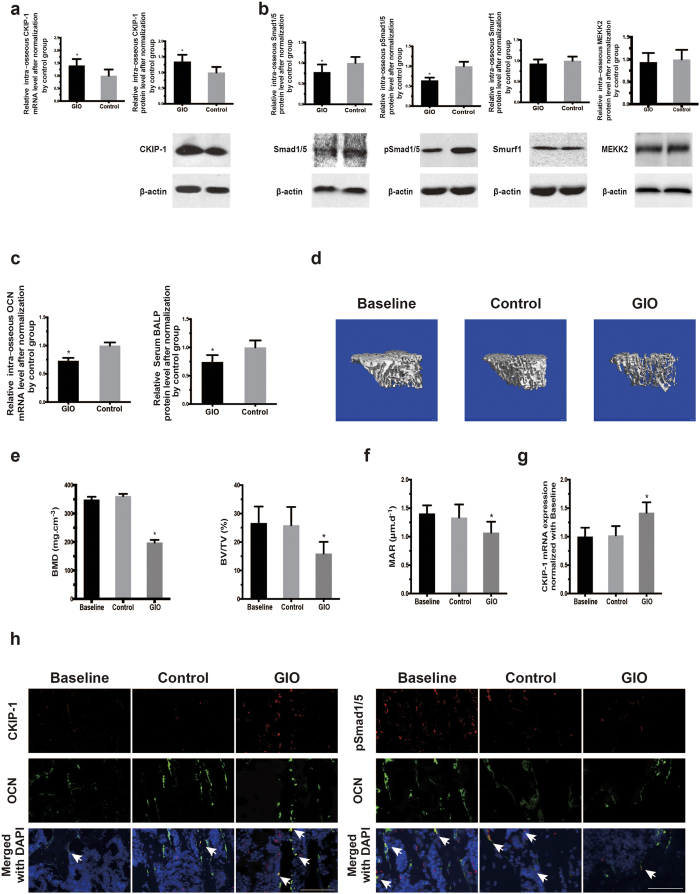
Highly expressed CKIP-1 together with downregulated Smad-dependent BMP signaling and decreased bone formation in GIO. (**a**) The intra-osseous CKIP1 mRNA (left) and protein (right) levels in patients from GIO and Control groups, respectively. (**b**) The intra-osseous total Smad1/5, pSmad1/5, Smurf1 and MEKK2 protein in patients from GIO and Control groups. Upper pannel: the quantitative data. Lower pannel: the representative electrophoretic bands (cropped). (**c**) The intra-osseous OCN mRNA level and the serum BALP level in patients from GIO and control groups. (**d**) Representative reconstructed micro-CT images for the trabecular architecture at the left tibia from the mice in each group. Scale bar: 100um. (**e**) The values of micro-CT parameters (BMD, BV/TV) in each group. (**f**) The values of bone histomorphometric parameter (MAR) in mice from each group. (**g**) The intra-osteoblast CKIP-1 mRNA levels in OCN + cells isolated by LCM in each group. (**h**) Representative immunofluorescence images for the expression of CKIP-1 (left three panel) and p-Smad1/5 (right three panel) in OCN + cells at the cryosection of proximal tibia from the mice in each group. Merged images with DAPI staining showed cells co-staining of CKIP-1 and OCN (arrow, left three lower panel), and p-Smad1/5 and OCN (arrow, right three lower panel). Scale bar = 50 μm. **Note**: Full-length electrophoretic bands are presented in Supplementary Figure 6. All data are the mean ± s.d. In the study with human specimens, the relative mRNA and protein levels are normalized to the mean value of the Control group. Human *GAPDH* mRNA and β-actin protein are used as the internal controls. LCM: the laser captured micro-dissection. In the study with mice specimens, mice *Gapdh* mRNA is used as the internal controls. *P < 0.05 for GIO group vs Control group.

To compare the differences in the levels of intra-osseous CKIP-1 and Smad-dependent BMP signaling as well as bone formation markers between the fracture patients with and without GIO, we collected iliac bone specimens and serum from 10 fracture patients with GIO and another 10 fracture patients without GIO (Supplementary Table 1). Real-time PCR and western blot analysis showed that the CKIP-1 mRNA and protein expression in bone specimens were both significantly higher in the GIO group than those in the control group (Fig. 1a). In contrast, the total Smad1/5 and pSmad1/5 protein expression in bone specimens were significantly lower in the GIO group than those in the control group (Fig. 1b). Unexpectedly, we found no statistical significant difference in the protein expression of intraosseous MEKK-2, another downstream substrate of CKIP-1-mediated ubiquitination, between the GIO and control groups (Fig. 1b). Similarly, the Smurf1 protein expression were alike between the GIO and control groups (Fig. 1b). Moreover, real-time PCR analysis and enzyme-linked immuno sorbent assay (ELISA) confirmed that the intra-osseous bone formation marker gene osteocalcin (OCN, a bone formation marker gene) and the serum bone formation marker bone-specific ALP (BALP, a circulating bone formation marker) were both lower in the GIO group than those in the control group (Fig. 1c).

We further examined the expression of CKIP-1 and p-Smad1/5 within osteoblasts during bone formation reduction in a mouse model of GIO. Sixteen 6-month-old male C57BL/6 mice were randomly divided into GIO group and Control group. Then, the mice were administered with either vehicle (Control Group, n = 8) or 2.1 mg/kg/day of prednisolone (GIO Group, n = 8) by subcutaneous implantation of slow release pellets for 30 days^16^. Another eight six-month-old male C57BL/6 mice were sacrificed before treatment as baseline. The micro CT analysis confirmed that the bone mineral density (BMD), relative bone volume (BV/TV), trabecular thickness (Tb.Th) and trabecular number (Tb.N) in the GIO group were significantly decreased from baseline and obviously lower than those in the Control group after 12 weeks of glucocorticoid treatment (Fig. 1d,e and Supplementary Figure 2a). The bone histomorphometric analysis further showed that mineral apposition rate (MAR) and osteoblasts surfaces (Ob.s/BS) in the GIO group were both significantly decreased from baseline and lower than those in the Control group (Fig. 1f and Supplementary Figure 2b,c). On the other hand, number of osteoclasts (N.Oc/BS) and osteoclasts surfaces (Oc.S/BS) in the GIO group were both significantly increased from baseline and higher than those in the Control group (Supplementary Figure 2d).Moreover, the laser captured micro-dissection (LCM) in combination with real time-PCR analysis revealed that the level of CKIP-1 mRNA expression in OCN + cells (osteoblasts) in the GIO group was significantly increased from baseline and higher than that in the Control group (Fig. 1g and Supplementary Figure 2e). Correspondingly, the immunofluorescence analysis showed that more instances of co-localization of CKIP-1 with osteocalcin positive staining (OCN + ) cells in the GIO group than those in the Control group and Baseline group, whereas less instances of co-localization of p-Smad1/5 with OCN + cells were found in the GIO group than those in the Control group and Baseline group (Fig. 1h). Collectively, the findings from human and mice concordantly suggested that the highly expressed CKIP-1 within osteoblasts was associated with the downregulated Smad-dependent BMP signaling and decreased bone formation in GIO.

### Highly expressed CKIP-1 inhibits Smad-dependent BMP signaling pathway to suppress osteogenic differentiation and mineral deposition in osteoblasts during glucocorticoid treatment *in vitro*

**Figure 2:**
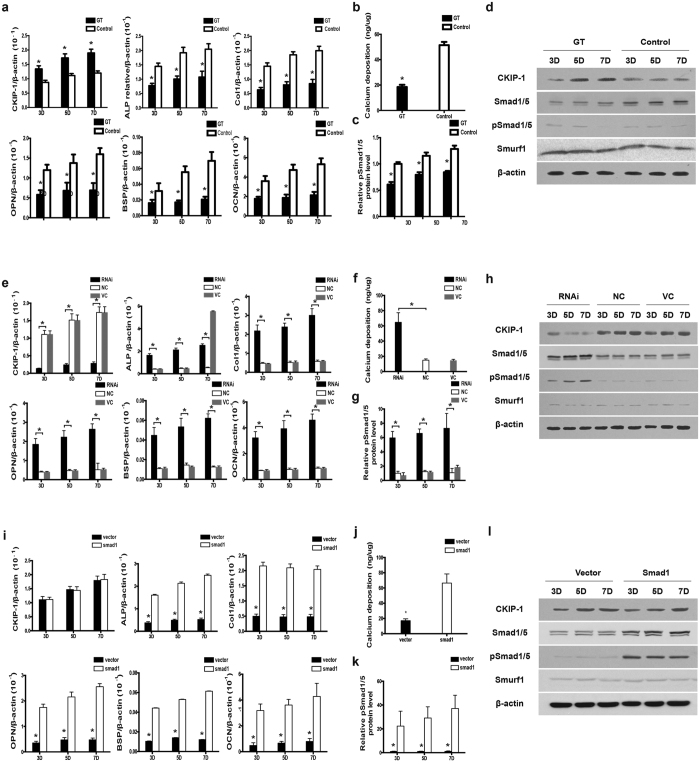
The effects of CKIP-1 knockdown and Smad1 overexpression on osteogenic activity and Smad-dependent BMP signaling in glucocorticoid-treated MC3T3-E1 cells *in vitro*. (**a**) The mRNA expression levels of CKIP-1 and osteogenic marker genes (ALP, COL1, OPN, BSP and OCN) in GT and Control groups at day 3, 5 and 7, respectively. (**b**) The amount of deposited calcium in GT and Control groups (left) at day 7, respectively. (**c**) Quantification of pSmad1/5 protein levels in GT and Control groups at day 3, 5 and 7, respectively. (**d**) Representative electrophoretic bands on the protein expression of CKIP-1, pSmad1/5, Smad1/5 and Smurf1 in GT and Control groups at day 3, 5 and 7, respectively. (**e**) The mRNA expression levels of CKIP-1 and osteogenic marker genes (ALP, COL1, OPN, BSP and OCN) in RNAi, VC and NC groups at day 3, 5 and 7, respectively. (**f**) The amount of deposited calcium in RNAi, VC and NC groups (left) at day 7, respectively. (**g**) Quantification of pSmad1/5 protein levels in RNAi, VC and NC groups at day 3, 5 and 7, respectively. (**h**) Representative electrophoretic bands on the protein expression of CKIP-1, pSmad1/5, Smad1/5 and Smurf1 in RNAi, VC and NC groups at day 3, 5 and 7, respectively. (**i**) The mRNA expression levels of CKIP-1 and osteogenic marker genes (ALP, COL1, OPN, BSP and OCN) in Smad1 and Vector groups at day 3, 5 and 7, respectively. (**j**) The amount of deposited calcium in Smad1 and Vector groups (left) at day 7, respectively. (**k**) Quantification of pSmad1/5 protein levels in Smad1 and Vector groups at day 3, 5 and 7, respectively. (**l**) Representative electrophoretic bands on the protein expression of CKIP-1, pSmad1/5, Smad1/5 and Smurf1 in Smad1 and Vector groups at day 3, 5 and 7, respectively. **Note**: All data were mean ± s.d. *p < 0.05 for GT group *vs.* Control group, RNAi group *vs*. NC and VC group or Smad1 group *vs.* Vector group in the corresponding study.

To investigate the molecular mechanism underlying the association between highly expressed CKIP-1 and downregulated Smad-dependent BMP signaling pathway in osteoblasts during the development of GIO, we first examined the effect of GC treatment on osteogenic differentiation and mineral deposition in MC3T3-E1 cells *in vitro*. Mouse osteoblasts-like MC3T3-E1 cells were treated with dexamethasone (10^−6^ M) (GT group) and the corresponding vehicle (Control group) for 7 days, respectively. We measured the mRNA expression of CKIP-1, alkaline phosphatase (ALP), collagen type I (COL1), osteopontin (OPN), bone sialoprotein (BSP) and osteocalcin (OCN) by quantitative real-time PCR at day 3, 5 and 7 (Supplementary Figure 3a). The CKIP-1 mRNA expression was significantly higher, whereas the mRNA expression of osteogenic marker genes (ALP, COL1, OPN, BSP and OCN) was lower in GT group than Control group at day 3, 5 and 7 during GC treatment (P < 0.05), respectively (Fig. 2a). We also quantified the matrix mineralization by Alizarin Red S calcium staining at day 7. The formation of calcium deposition was observed at day 7 in both groups but the amount of deposited calcium was much lower in GT group than Control group (P < 0.05) (Fig. 2b). Furthermore, we determined the protein expression of CKIP-1, Smad1/5, pSmad1/5 and Smurf1 as well as the ubiquitination level of Smad1 by western blotting at day 3, 5 and 7. The CKIP-1 protein expression was higher, while the protein expression of pSmad1/5 and Smad1/5 were both lower in GT group than Control group at day 3, 5 and 7 (P < 0.05), respectively (Fig. 2c,d, and Supplementary Figure 3b). The Smurf1 protein expression remained unchanged during GC treatment (Fig. 2d). Moreover, the ubiquitination level of Smad1 was also higher in GT group than Control group at day 3, 5 and 7, respectively (Supplementary Figure 3c).

We next examined the effects of CKIP-1 knock down by siRNA on osteogenic differentiation and mineral deposition in MC3T3-E1 cells treated with GC *in vitro*, the MC3T3-E1 cells were transfected with CKIP-1 siRNA encapsulated within Lipofectamine™ 2000 (RNAi Groups), non-sense siRNA encapsulated within Lipofectamine™ 2000 (NC Group) and Lipofectamine™ 2000 only (VC Group), respectively. All the groups were treated with GC for 7 days (Supplementary Figure 3d). After treatment with CKIP-1 siRNA, the CKIP-1 mRNA expression was significantly down-regulated, while the mRNA expressions of osteogenic marker genes were all notably up-regulated, when compared to NC and VC groups at day 3, 5 and 7 during GC treatment (P < 0.05), respectively (Fig. 2e). The amount of deposited calcium was much higher in RNAi group than NC and VC groups at day 7 (P < 0.05) (Fig. 2f). Correspondingly, the RNAi Group showed significantly lower CKIP-1 protein expression but higher pSmad1/5 and Smad1/5 when compared to NC and VC groups at day 3, 5 and 7, respectively (Fig. 2g,h and Supplementary Figure 3e). No obvious differences in the Smurf1 protein expression were observed among the three groups at each time point (P < 0.05) (Fig. 2h). In addition, the RNAi Group showed lower ubiquitination level of Smad1 when compared to NC and VC groups at each time point, respectively (Supplementary Figure 3f).

Further, we investigated whether the highly expressed CKIP-1 could inhibit Smad-dependent BMP signaling pathway to suppress osteogenic differentiation and mineral deposition in MC3T3-E1 cells treated with GC *in vitro*. The MC3T3-E1 cells were transfected with either lentivirus vector pGLV-U6-GFP encoding Smad1 mRNA (Smad1 Group) or blank lentivirus vector (Vector Group) (Supplementary Figure 3g). Then, the cells in the two groups were treated with GC as described above. We found that, despite the high CKIP-1 mRNA expression in both groups, the osteogenic genes mRNA expressions were constantly higher in Smad1 Group than Vector Group at day 3, 5 and 7 (P < 0.05) during GC treatment, respectively (Fig. 2i). Consistently, the amount of deposited calcium was much higher in Smad1 Group than Vector Group (P < 0.05) (Fig. 2j). Although no significant difference in CKIP-1 protein expression was found between the two groups, the expression of pSmad1/5 and Smad1/5 protein were both higher in Smad1 Group than Vector Group at day 3, 5 and 7 (P < 0.05), respectively (Fig. 2k,l and Supplementary Figure 3h). Taken together, these results suggested that highly expressed CKIP-1 could inhibit the Smad-dependent BMP signaling pathway to suppress osteogenic differentiation and mineral deposition in osteoblasts during GC treatment *in vitro.*

### Ablation of Ckip-1 in osteoblasts prevents bone formation reduction during GIO development in mice

**Figure 3:**
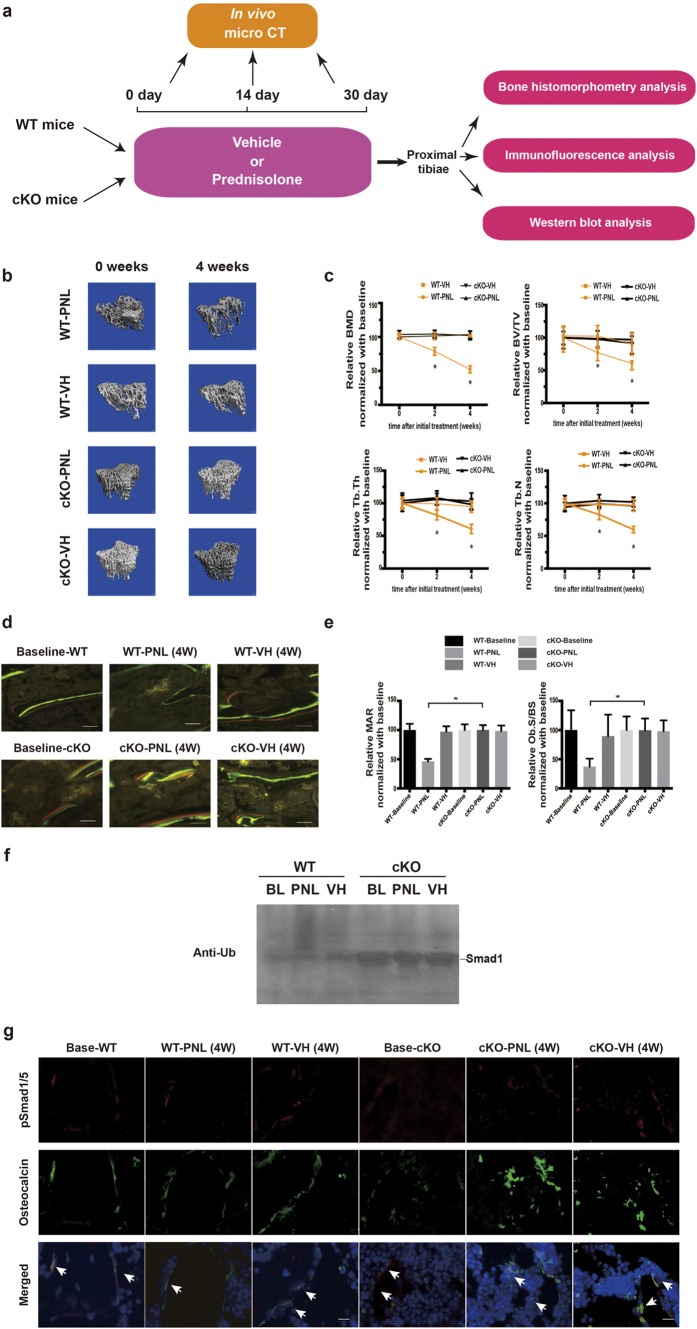
The effects of genetic ablation of *Ckip-1* within osteoblast on bone formation and Smad-dependent BMP signaling in glucocorticoid-treated mice. (**a**) Schematic diagram of the experimental design. (**b**) Representative *in vivo* micro-CT images of the time-course changes in three-dimensional trabecular architecture of the left proximal tibiae from each group. Scale bar: 100 um. (**c**) The time-course changes in micro-CT parameter (BMD) of the left proximal tibia from each group. (**d**) Representative micrographs of newly mineralized bone assessed by xylenol (red) and calcein (green) labeling at the left proximal tibiae from each group after 4 weeks. Scale bar: 50 μm. (**e**) The changes in the bone histomorphometric parameter (MAR and Ob.S/BS) of the left proximal tibiae from each group after 4 weeks. (**f**) The representative electrophoretic bands of the time-course changes in the ubiquitination of total Smad1 in bone tissue level from each group. The mice were treated with MG132 (2 mg/kg) through intraperitoneal injection 24 hours before sample collections. Polyubiquitinated Smad1 was detected by anti-ubiquitin immunoblot analysis after precipitation of Smad1 in the cell lysis of MC3T3-E1 cells pretreated with proteasome inhibitor MG132. (**g**) The representative immunofluorescence images for the time-course changes in the co-expression of p-Smad1/5 (red) and OCN + (green) cells of the right proximal tibiae from each group. Merged images with DAPI staining showed cells co-staining of p-Smad1/5 with Osteocalcin (arrow indicated). Scale bar: 25 μm. **Note:** The data were mean ± s.d. *P < 0.05 for WT-PNL group vs cKO-PNL group. WT: wildtype littermate mice; cKO: osteoblast-specific *Ckip-1* knockout mice. Base: Baseline of before glucocorticoid treatment. PNL: prednisolone treatment; VH: Vehicle treatment.

To test the effect of genetic ablation of Ckip-1 in osteoblasts on bone formation during GIO development in mice, we generated the osteoblasts-specific Ckip-1-knockout (cKO) mice by crossing Ckip-1^*fl/fl*^ mice with Osx-Cre mice (Supplementary Figure 4a–d). The littermate mice were used as wild-type (WT) controls. Six-month-old male cKO mice and WT mice were administered with either vehicle (cKO-VH Group, n = 8; WT-VH Group, n = 8) or 2.1 mg/kg/day of prednisolone (cKO-PNL Group, n = 8; WT-PNL Group, n = 8) by subcutaneous implantation of slow release pellets for 30 days as aforementioned. Another eight 6-month-old male cKO mice and eight WT mice were sacrificed before GC treatment as baselines (Fig. 3a). There was no observed significant difference in body weight either between cKO mice at baseline and cKO mice after GC treatment, or between GC-treated cKO mice and GC-treated controls (Supplementary Table 2). Considering that the skeletal phenotype was different between the cKO mice and their littermate control at baseline, we adjusted the values of micro-CT and bone histomorphometric parameters of each group after GC treatment to the values at baseline before GC treatment to eliminate the bias in bone phenotypes at baseline (Supplementary Table 3). The *in vivo* micro-CT analysis showed progressive deterioration in trabecular microarchitecture in WT-PNL group when compared to that in WT-VH group (Fig. 3b). However, the trabecular microarchitecture was almost preserved in cKO-PNL group as compared to that in cKO-VH group (Fig. 3b). Consistently, the micro-CT parameters (BMD, BV/TV, Tb.Th and Tb.N) were all decreased gradually from baseline in WT-PNL group but maintained in the other three groups (Fig. 3c). The Bone histomorphometry analysis further demonstrated that, after 4 weeks of GC treatment, the MAR and Ob.S/BS both notably decreased from baseline in WT-PNL Group, whereas they were both maintained in WT-VH, cKO-PNL and cKO-VH Groups (Fig. 3d, e and Supplementary Figure 4e). Moreover, N.Oc/BS and Oc.S/BS were significantly higher in glucocorticoid-treated groups when compared with non-glucocorticoid control groups. However, we found no significant difference in N.Oc/BS and Oc.S/BS among glucocorticoid-treated groups (Supplementary Figure 4f). Furthermore, the immunoblotting analysis revealed that the ubiquitination level of total Smad1 in OCN^+^ bone marrow cells in WT-PNL group was notably lower than WT-VH group. No obvious difference in the ubiquitination level of total Smad1 in whole bone tissues were observed between cKO-PNL and cKO-VH groups (Fig. 3f). In addition, the immunofluorescence analysis showed obviously fewer instances of cells co-expressing p-Smad1/5 and OCN in WT-PNL group when compared to those in WT-VH group, whereas the above difference was not detected between cKO-PNL and cKO-VH groups (Fig. 3g). These findings suggested that the bone formation reduction during GIO development in mice could be prevented by the genetic ablation of CKIP-1 in osteoblasts.

### Overexpressing *Smad1* in osteoblasts attenuates bone formation reduction during GIO development in mice

**Figure 4:**
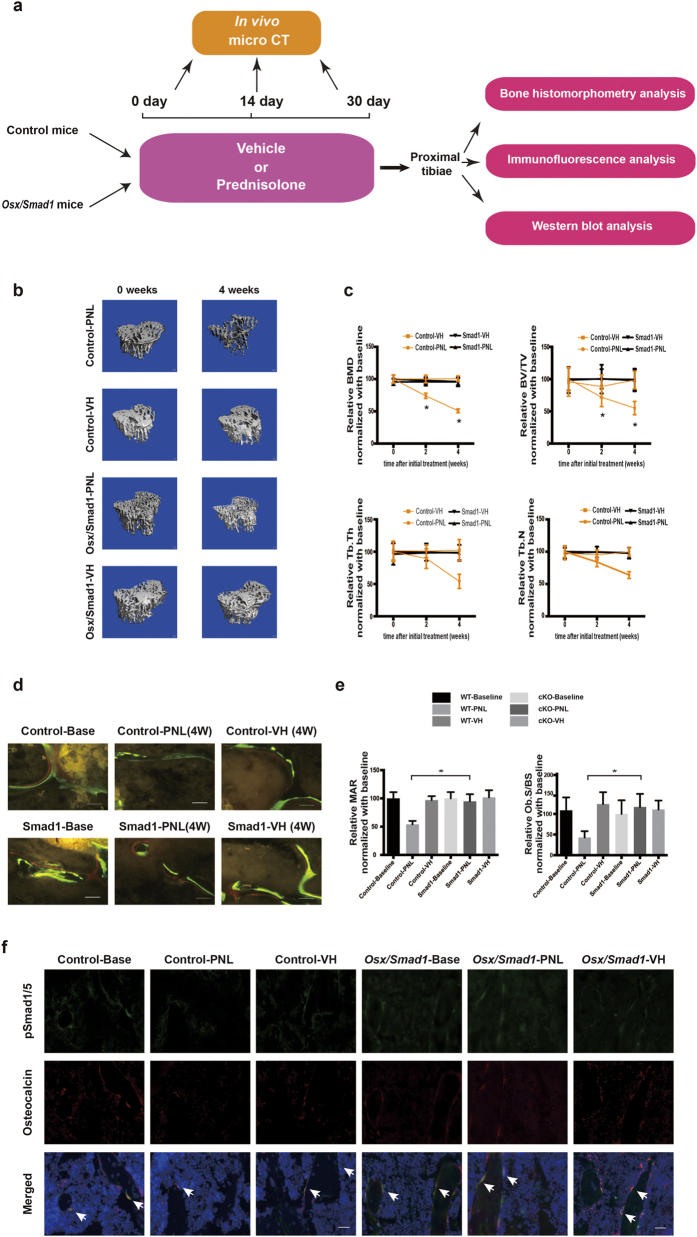
The effects of genetic overexpression of *Smad1* within osteoblast on bone formation and Smad-dependent BMP signaling in glucocorticoid-treated mice. (**a**) Schematic diagram of the experimental design. (**b**) Representative *in vivo* micro-CT images of the time-course changes in three-dimensional trabecular architecture of the left proximal tibiae from each group. Scale bar: 100 um. (**c**) The time-course changes in micro-CT parameter (BMD) of the left proximal tibia from each group. (**d**) Representative micrographs of newly mineralized bone assessed by xylenol (red) and calcein (green) labeling at the left proximal tibiae from each group after 4 weeks. Scale bar: 50 μm. (**e**) The changes in the bone histomorphometric parameter (MAR and Ob.S/BS) of the left proximal tibiae from each group after 4 weeks. (**f**) The representative immunofluorescence images for the time-course changes in the co-expression of p-Smad1/5 (green) and OCN + (red) cells of the right proximal tibiae from each group. Merged images with DAPI staining showed cells co-staining of p-Smad1/5 with Osteocalcin (arrow indicated). Scale bar: 25 μm. **Note:** The data were mean ± s.d. *P < 0.05 for Control-PNL group vs *Osx/Smad1*-PNL group. Control: *Osx-Cre* mice; *Osx/Smad1*: osteoblast-specific *Smad1* knock-in mice. Base: Baseline of before glucocorticoid treatment. PNL: prednisolone treatment; VH: Vehicle treatment.

We next investigated whether conditionally overexpressing *Smad1* in osteoblasts could reverse the inhibitory effect of CKIP-1 on the bone formation during GIO development in mice. To this end, we generated the osteoblasts-specific Smad-1-overexpressed mice (Supplementary Figure 5a–d) and administered them with either vehicle (*Osx/Smad1*-VH Group, n = 8; Control-VH Group, n = 8) or 2.1 mg/kg/day of prednisolone (*Osx/Smad1*-PNL Group, n = 8; Control-PNL Group, n = 8) by subcutaneous implantation of slow release pellets for 30 days ^[8]^. Another eight 6-month-old male *Osx/Smad1* mice and eight control mice were sacrificed before GC treatment as baselines (Fig. 4a). No significant difference was found in body weight either between *Osx/Smad1* mice at baseline and *Osx/Smad1* mice after GC treatment, or between GC-treated *Osx/Smad1* mice and GC-treated controls (Supplementary Table 2). The skeletal phenotype was also different between the *Osx/Smad1* mice and their littermate control at baseline, thus we adjusted the values of micro-CT and bone histomorphometric parameters of each group after GC treatment to the values at baseline before GC treatment to eliminate the bias in bone phenotypes at baseline (Supplementary Table 3).The *in vivo* micro-CT analysis revealed that the trabecular microarchitecture was progressively deteriorated in Control-PNL group when compared to that in Control-VH group. However, the above deterioration in trabecular microarchitecture was attenuated in *Osx/Smad1*-PNL group when compared to *Osx/Smad1*-VH group (Fig. 4b). Consistently, the micro-CT parameters were all decreased continuously from baseline in Control-PNL group when compared to those in Control-VH group. In comparison, the above decreases in the micro-CT parameters were obviously alleviated in *Osx/Smad1*-PNL group when compared to *Osx/Smad1*-VH group (Fig. 4c). Similarly, the bone histomorphometry analysis demonstrated that the MAR and BFR/BS were both notably decreased from baseline in Control-PNL Group when compared to those in Control-VH group, whereas the above decrease in MAR and Ob.S/BS were notably attenuated in *Osx/Smad1*-PNL group when compared to *Osx/Smad1*-VH group (Fig. 4d,e and Supplementary Figure 5e). N.Oc/BS and Oc.S/BS were significantly higher in glucocorticoid-treated groups when compared with non-glucocorticoid control groups. No significant difference was found in N.Oc/BS and Oc.S/BS among glucocorticoid-treated groups (Supplementary Figure 5f). Furthermore, the immunofluorescence analysis showed more instances of cells co-expressing p-Smad1/5 and OCN in *Osx/Smad1*-PNL group when compared to those in Control-PNL group (Fig. 4f). These findings suggested that the bone formation reduction during GIO development in mice could be attenuated by the genetic overexpression of Smad1 in osteoblasts.

### Osteoblasts-targeting CKIP-1 siRNA treatment attenuates bone formation reduction during GIO development

**Figure 5:**
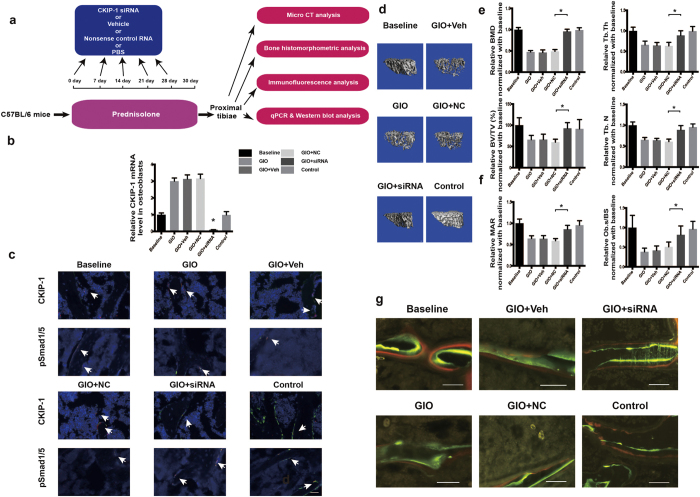
The effect of therapeutic inhibition of CKIP-1 within osteoblast on Smad-dependent BMP signaling and bone formation in glucocorticoid-treated mice. (**a**) Schematic diagram of the experimental design. (**b**) The level of CKIP-1 mRNA expression in OCN + cells isolated by LCM from cryosection of proximal tibia in each group. (**c**) Representative immunofluorescence images for CKIP-1 and pSmad1/5 expression in OCN + cells in cryosection of proximal tibia from each group. Upper panel: CKIP-1, green; OCN, red; Nucleus, blue. Lower panel: pSmad1/5, green; OCN, red; Nucleus, blue. Scale bars: 25 μm. (**d**) Representative images for three-dimensional trabecular architecture at proximal tibia from each group. Scale bars: 100 μm. (**e**) Micro-CT parameters for bone mass (BMD, BV/TV) and trabecular architecture (Tb.Th and Tb.N) at proximal tibia from each group. (**f**) Bone histomorphometric parameters (MAR and Ob.S/BS) at proximal tibia from each group. (**g**) Representative fluorescence microscopic images of fluorescence-labeling with xylenol orange (red)/calcein green (green) of the left proximal tibiae from each group. Scale bar: 50 um. **Note:** All data were mean ± s.d. *P < 0.05. n = 8 mice in each group. Baseline: mice sacrificed before glucocorticoid treatment, GIO: mice administrated with phosphate buffer solution (negative control), GIO + Veh: mice administrated with delivery system only, GIO + NC: mice administrated with nonsense RNA negative control with delivery system, GIO + siRNA: mice administrated with CKIP-1 siRNA encapsulated within osteoblast-targeting delivery system, Control: mice that were not treated with glucocorticoid.

We further tested whether therapeutic inhibition of CKIP-1 within osteoblasts *in vivo* could also attenuate bone formation reduction during GIO development. Thirty-two 6-month-old male C57BL/6 mice were administered with 2.1 mg/kg/day of prednisolone (GIO Group, n = 8) by subcutaneous implantation of slow release pellets for 30 days and were thereafter sacrificed. Meanwhile, the above mice were randomly divided into the GIO + siRNA group, GIO + Veh group, GIO + NC group and GIO group (n = 8 for each group) and received four consecutive intravenous injections of CKIP-1 siRNA (7.5 mg/kg) encapsulated in our previously reported osteoblasts-targeting delivery system^17^, osteoblasts-targeting delivery system only (vehicle), nonsense control (NC) RNA encapsulated in osteoblasts-targeting delivery system or PBS, respectively, at an interval of 7 days. Another eight age-matched male mice were sacrificed before GC treatment as baseline. On the other hand, eight more male mice remained untreated for 30 days and sacrificed as age-matched control (Fig. 5a). Real-time PCR and immunofluorescence analysis showed that, as compared to baseline group, both mRNA and protein expression of CKIP-1 within osteoblasts (OCN + cells) were significantly higher and pSmad1/5 level within osteoblasts was significantly lower in GIO, GIO + NC and GIO + Veh groups, respectively (Fig. 5b,c). However, the GIO + siRNA group exhibited lower levels of intra-osteoblasts CKIP-1 mRNA and protein and higher level of intra-osteoblasts pSmad1/5 as compared to GIO, GIO + NC and GIO + Veh groups (Fig. 5b,c). As shown by micro-CT analysis of proximal tibia, the trabecular bone mass was markedly reduced (lower in BMD and BV/TV) and the trabecular architecture was impaired (lower in Tb.Th and Tb.N) in GIO, GIO + NC and GIO + Veh groups when compared to baseline group, whereas the reduced trabecular bone mass and deteriorated trabecular architecture was attenuated in GIO + siRNA group as compared to GIO, GIO + NC and GIO + Veh groups (Fig. 5d,e). Moreover, bone histomorphometric analysis of proximal tibia also revealed that MAR and Ob.S/BS were both lower in GIO, GIO + NC and GIO + Veh groups when compared to Baseline group, whereas the above parameters were higher in GIO + siRNA group when compared to GIO, GIO + NC and GIO + Veh groups (Fig. 5f,g and Supplementary Figure 6a). Consistently, no significant difference was found in N.Oc/BS and Oc.S/BS among glucocorticoid-treated groups (Supplementary Figure 5f). Collectively, these results demonstrated that osteoblasts-targeting CKIP-1 siRNA treatment could also attenuate the bone formation reduction during GIO development in mice.

## Discussion

This is the first study to elucidate the role of ubiquitination-related molecule CKIP-1 within osteoblasts in regulating the Smad-dependent BMP and bone formation during the development of GIO.

In the current study, we examined the bone specimens from GIO patients and found the highly expressed CKIP-1 and lowly expressed pSmad1/5 at whole bone tissue level together with the reduced level of bone formation markers in bone and serum. In GIO mouse model, we further observed an increase expression of intra-osteoblasts CKIP-1 and a decrease in intra-osteoblasts pSmad1/5 and reduction in bone formation during GIO. The above findings concordantly suggest a close association between the aberrantly high expression of CKIP-1 in osteoblasts with the repressed Smad-dependent BMP signaling pathway and reduced bone formation under the pathophysiological condition of GIO. Consistently, we observed that the overdose GC could induce the expression of CKIP-1 and suppress the Smad-dependent BMP signaling, osteogenic differentiation and mineral deposition in MC3T3-E1 cells. Furthermore, the above inhibitory effects of GC on Smad-dependent BMP signaling and osteogenic activity as well as bone formation were prevented by siRNA-mediated CKIP-1 knockdown in MC3T3-E1 cells *in vitro* or genetic ablation of osteoblastic *Ckip-1* in cKO mice *in vivo*, respectively. Taken together, it suggests that the aberrantly high CKIP-1 in osteoblasts could contribute to the repressed Smad-dependent BMP signaling and reduced bone formation in GIO.

It has been previously reported that CKIP-1 could facilitate the Smurf1-mediated ubiquitination of Smad1/5 and MEKK-2 to regulate canonical Smad-dependent BMP signaling pathway and noncanonical BMP-JNK signaling pathway, respectively^13^. Although several previous studies demostrated that GC treatment could significantly suppress the Smad-dependent BMP signaling pathway in osteoblasts^14^,^15^, there is no study reporting the involvement of dysregulated MEKK-2 and the downstream pathway in osteoblasts during the development of GIO. Intriguingly, our data from human bone specimens showed no obvious difference in the MEKK-2 protein expression between GIO and control groups, suggesting that the upregulated CKIP-1 did not result in notable decrease in MEKK-2 protein levels under the pathological condition of GIO. Recently, it has been reported that Cdh1, another ubiquitination-related molecule that could also augment Smurf1 activity^18^,^19^, rather than CKIP-1 has a pivotal role in regulating the MEKK-2 pathway. Moreover, another recent study observed the unaltered BMP response in *Mekk2* gene-depleted osteoblasts and demonstrated that MEKK-2 is dispensable for noncanonical BMP-JNK pathway activation in osteoblasts^20^. Thus, we focused on the CKIP-1-mediated ubiquitination regulation of Smad-dependent BMP signaling within osteoblasts during the development of GIO. In the present study, we found that the high CKIP-1 expression was accompanied by a low expression of total Smad1/5 in bone samples from GIO patients. We further observed that the increase in CKIP-1 expression was accompanied by a decrease in total Smad1/5 expression and an increase in Smad1 ubiquitination in MC3T3-E1 cells after GC treatment, suggesting that the aberrant ubiquitination-related regulatory mechanism may contribute to the repressed Smad-dependent BMP signaling in GC-inhibited osteoblasts. Interestingly, we found no significant differences in the mRNA and protein expression of Smurf1 in bone specimens as well as in MC3T3-E1 cells between GIO and control groups, suggesting that CKIP-1 could make greater contribution to the increased Smad1 ubiquitination and repressed Smad-dependent BMP signaling than Smurf1 in GC-inhibited osteoblasts. More importantly, we found that the increased Smad1 ubiquitination in GC-treated osteoblasts as well as in bone specimens from GIO mice was prevented after siRNA-mediated knockdown and genetic ablation of *Ckip-1* in osteoblasts, respectively. In addition, the above inhibitory effects of GC on Smad-dependent BMP signaling, osteogenic activity and bone formation could be attenuated by genetic overexpression of osteoblastic *Smad1* either in MC3T3-E1 cells *in vitro* or in *Osx/Smad1* mice *in vivo*. Collectively, it suggests that the aberrantly high CKIP-1 in osteoblasts could promote the Smad1 ubiquitination to dampen the Smad-dependent BMP signaling and contribute to the reduced bone formation in GIO.

Apart from the continually decreased osteoblastic bone formation, recent study has shown that osteoclastic bone resorption could be transiently increased after GC treatment^21^. In this study, we consistently found that GC could also induce osteoclast bone resorption^22^,^23^,^24^. Moreover, CKIP-1 has been reported to be an important inhibitor of macrophage proliferation and thus could be involved in molecular mechanisms responsible for osteoclastic bone resorption^25^. However, previous study demonstrated that osteoclastic bone resorption was not changed in CKIP-1 systemic knockout mice as compared to wild type controls^13^. In our study, we did not observe significant difference in osteoclastic bone resorption between CKO mice and corresponding control mice after GC treatment, indicating that osteoblastic CKIP-1 might not affect osteoclastic bone resorption.

On the other hand, a previous study has documented that recombinant human BMP-2 (rhBMP-2) could not completely rescue the GC-inhibited osteoblasts phenotype^26^, again highlighting the importance of intervening the downstream inhibitory factors, such as ubiquitination-related regulatory mechanism, during BMP signaling transduction in addition to supplement of BMPs for recovering the BMP signaling and osteogenic activity in GC-inhibited osteoblasts. In the current study, we delineated a novel mechanism involving CKIP-1-mediated ubiquitination regulation of Smad-dependent BMP signaling responsible for the reduced bone formation in GIO. Furthermore, we demonstrated that osteoblasts-targeting CKIP-1 siRNA treatment could significantly enhance the Smad-dependent BMP signaling, increase bone mass and improve trabecular microarchitecture to attenuate the bone formation reduction in GIO mice, suggesting that the osteoblastic CKIP-1 could be a promising therapeutic target for GIO.

In conclusion, we demonstrated that the highly expressed CKIP-1 in osteoblasts could suppress the Smad-dependent BMP signaling and contribute to the bone formation reduction in GIO, suggesting that targeting osteoblastic CKIP-1 would be a novel bone anabolic strategy for GIO patients.

## Materials and Methods

### Human bone specimen preparation

We collected bone specimens from 10 fracture patients with GIO and another 10 fracture patients without GIO. They were all diagnosed as intertrochanteric fractures and admitted in the Xijing Hospital between Jan 2013 and Oct 2014 to receive fracture surgery of internal fixation and autogenous iliac bone-grafting (Supplementary Table 1). Informed consent for the use of surgically removed tissue and serum was obtained from the patient before surgery. The iliac bone specimens and serum from each patient were simultaneously harvested during surgery and cryopreserved and were stored in liquid nitrogen for further mRNA and protein analysis, respectively. All experiments were performed in accordance with relevant guidelines and regulations and all clinical procedures were approved by the Ethics Committees of the Xijing Hospital, Shenzhen 8th People Hospital and Shenzhen People’s Hospital, respectively.

### Animal model

The osteoblasts-specific Ckip-1 knockout (cKO) mice were generated by crossing the *Ckip-1*^*fl/fl*^ mice with *Osx-Cre* mice. The osteoblasts-specific Smad1 knock-in (*Osx/Smad1*) mice were obtained by crossing the mice containing the ROSA26-PCAG-STOP^*fl*^-*Smad1* knock-in allele with *Osx-Cre* mice. The C57BL/6 and genetically-modified mice were all maintained under standard animal housing conditions (12-h light, 12-h dark cycles and free access to food and water). For GIO model, mice were subcutaneously implanted with slow-release prednisone pellets (Innovative Research of America, Sarasota, Florid, USA) containing 5 mg of prednisolone or vehicle^16^,^23^. A dosage of 2.1 mg/kg/day prednisolone was estimated to be delivered by the slow-release pellets. Body weight was measured at 1 day before (baseline) and 28 days after pellet implantation. At the corresponding time points in each study, the mice were euthanized for sample collection. All experiments were performed in accordance with relevant guidelines and regulations and all experimental procedures were approved by the Committees of Animal Ethics and Experimental Safety of Hong Kong Baptist University and the Chinese University of Hong Kong.

### Total RNA extraction, reverse transcription and quantitative real-time PCR

RNeasy Mini Kit (QIAGEN, Cat. No. 74106) was used to extract total RNA from cultured cells and tissues using the commercialized protocol. For cultured cells, after aspirating the medium, the cells were washed with PBS and trypsinized with 0.25% trypsin in PBS. Then, the full medium containing serum was added to inactivate the trypsin and the cells were transferred to an RNase-free polypropylene centrifuge tube and centrifuged at 300 × g for 5 minutes. After centrifugation, the cell pellet was disrupted by adding 350 μl buffer RLT and homogenized by pipetting up and down. For tissues, 600 μl of Buffer RLT were added directly to the tissues after crashed by pestle grinder. After centrifugation at 8000 × g (10000 rpm) for 5 minutes, the supernatant was collected. Then, one volume of 70% ethanol was added to the homogenized lysate, mixed well and transferred to an RNeasy spin column and spun for 15 seconds at 8000 × g (10,000 rpm). After discarded the flow-through, the spin column membrane was washed by adding 700 μl Buffer RW1 and centrifuged for 15 seconds at 8000 × g (10,000 rpm). Thereafter, the spin column membrane was washed twice by 500 μl Buffer RPE and centrifuged for 15 seconds at 8000 × g (10,000 rpm). Then, 50 μl RNase-free water was added directly to the spin column membrane. After centrifuged for 1 min at 8000 × g (10,000 rpm), the total RNA was eluted from the column. The yield and purity of RNA were measured by spectrophotometry.

Total RNA was reverse-transcribed to cDNA using the following established protocol. A total volume of 12 μl reaction volume, including 1 μg total RNA, 1 μl 50 ng/μl random primers and 1 μl dNTP Mix (10 mM each), was heated to 65 °C for 5 minutes and quick c hill on ice. And then, collected the contents of the tube was by brief centrifugation and add 4 μl 5X First-Strand Buffer, 2 μl 0.1 M DTT and 1 μl RNaseOUT™ (40 units/μl). Thereafter, mixed the contents in the tube and added 1 μl (200 units) of SuperScript™ II RT for mixture by pipetting gently up and down. After that, incubated the mixture at 42 °C for 50 minutes to get the cDNA and inactivate the reaction by heating at 70 °C for 15 minutes.

The 10 μl volume of the final quantitative real-time PCR solution contained 1 μl of the diluted cDNA product, 5 μl of 2X Power SYBR^®^ Green PCR Master Mix (Applied Biosystems, Foster City, California, U.S.A.), 0.5 μl each of forward and reverse primers and 3 μl nuclease-free water. The primer sequences were listed in Supplementary Table 1. The amplification conditions were as follows: 50 °C for 2 minutes, 95 °C for 10 minutes, 40 cycles of 95 °C for 15 secon ds, and 60 °C for 1 minute. The fluorescence signal emitted was collected by ABI PRISM^®^ 7900HT Sequence Detection System and the signal was converted into numerical values by SDS 2.1 software (Applied Biosystems). The mRNA expression level of the target gene was first calculated from the Relative Standard Curve Method by the SDS 2.1 software. The threshold cycle (CT) value, which represents the relative expression of each target gene, was determined from the corresponding curve. Then, the relative expression of mRNA was determined by dividing the target amount by endogenous control amount to obtain a normalized target value^27^.

### Immunoprecipitation

For Immunoprecipitation (IP), the MC3T3-E1 cells and femora bone samples were collected, respectively, and homogenized on ice with a Dounce homogenizer in IP lysis buffer and incubate on an orbital rotator at 4 °C for 24 hours. Then the homogenates were centrifugated at 12000 g for 10 min. After total protein quantification, the cell lysates were subjected to magnetic IP (Pierce Classic Magnetic IP/Co-IP Kit, Thermo Scientific) according to the manufacturer’s recommendation. Briefly, the cell lysates with a total of 500 ug protein were combined with 4 ug of anti-Smad1 antibodies (Abcam, ab131371), respectively, and incubated on an orbital rotator overnight at 4 °C to form the immune complex. Thereafter, the above antigen sample/antibody mixtures were added into a 1.5 mL microcentrifuge tube containing the pre-washed magnetic beads and incubated at room temperature for 1 hour with mixing. Then, the beads were collected with a magnetic stand while the supernatant with unbound antigens were saved as the Input for the downstream western blotting analysis. After that, the beads were washed twice with the IP wash buffer followed by another wash with ultrapure water. Then, the beads were mixed with the western blot sample buffer and heated at 99 °C in a heating block for 10 min for elution of the combined target antigen (Smad1). Finally, the supernatant-containing target antigen were magnetically separated from the beads and subjected to western blotting analysis^28^.

### Western blot analysis

Total proteins (40 μg) were loaded from each sample on denaturing SDS-PAGE. Immuno-detection of human and mouse CKIP-1, total Smad1/5 and phosphorylated Smad1/5 as well as rat Smurf1 were done using anti-CKIP-1 antibody (1: 500; Santa Cruz Biotechnology, USA), anti-Smad1/5 antibody (1:500; Santa Cruz Biotechnology, USA), anti-phosphorylated Smad1/5 antibody (1:1000; Cell Signaling Technology, Inc., USA) and anti-Smurf1 (1:500, Santa Cruz Biotechnology, USA), respectively. Enhanced chemiluminescence was used for detection. β-actin was used as control and detected by an anti-β-actin monoclonal antibody (1: 500; Sigma, USA). The relative amounts of the transferred proteins were quantified by scanning the auto-radiographic films with a gel densitometer (Bio-Rad, USA) and normalized to the corresponding β-actin level. For immuno-detection of the ubiquitination level of endogenous Smad1, the PVDF membrane was probed with the anti-ubiquitin antibody (1:500, Santa Cruz Biotechnology, USA).

### Laser Captured Micro-dissection (LCM)

The right tibias were decalcified in 10% EDTA and embedded in OCT. Then, the series frozen sections (5 μm) from proximal tibiae were prepared in a cryostat (CM3050; Leica Microsystems, Wetzlar, Germany) at −24 °C. The adjacent sections were mounted on either glass slides or polyethylene membrane–equipped slides (P.A.L.M., Bernried, Germany). The sections mounted on glass slides were performed immunofluorescence to identify the OCN-positive cells. Briefly, the cryosections were incubated overnight at 4 °C with rabbit polyclonal anti-OCN antibody (1:50 dilution; Santa Cruz Biotechnology, USA) after fixation and blocking. Then, the sections were incubated with Alexa Fluor 488-conjugated donkey anti-rabbit IgG (1:400 dilution; Invitrogen). Finally, the sections were mounted with medium containing DAPI (Vector laboratories) and examined under a fluorescence microscope to identify the OCN-positive staining cells. The adjacent sections mounted on membrane-coated slides were stained with neutral red for 1 min at room temperature. After brief rinsing in water, the sections were air-dried. OCN-positive staining cells in adjacent sections were isolated by microdissection with an upgraded laser pressure catapulting microdissection system (P.A.L.M.) using a pulsed 355 nm diode laser in the Leica LMD 7000 Laser Microdissection System. About 100~200 identified cells were collected in reaction tube containing 5 μl lysis buffer for total RNA extraction and subsequent real-time PCR analysis^29^.

### Micro-CT

For *in vivo* micro-CT analysis, the mice will be placed into a plastic cylindrical holder under anesthesia (75 mg/kg ketamine and 10 mg/kg xylazine) and scanned by the micro-CT system (viva CT40, SCANCO MEDICAL, Switzerland). The hind limb was positioned alone the scanning axis and the ankle was fixed to avoid movement during scanning. Totally slices with a voxel size of 21um was scanned at the region of proximal tibia beginning at the growth plate extending distally along the tibial diaphysis^17^,^29^. Eighty continuous slices beginning at 0.3 mm from the most distal aspect of the growth plat extending distally along the tibia diaphysis was selected for analysis. All the trabecular bone from each selected slice was segmented for 3D reconstruction (Sigma = 1.2, Supports = 2 and Threshold = 220) to calculate the following parameters: bone mineral density (BMD), relative bone volume (BV/TV), trabecular number (Tb.N), trabecular thickness (Tb.Th). For *ex vivo* micro-CT analysis, the tibia specimens were collected and scanned by the same micro-CT system. Briefly, a total of 416 slices with a voxel size of 15 μm was scanned at the region of proximal tibia beginning at the growth plate extending distally along the tibial diaphysis^27^. The whole trabecular bone was isolated for three-dimension reconstruction (Sigma = 1.2, Supports = 2 and Threshold = 220) to calculate BMD, BV/TV, Tb.N, Tb.Th.

### Bone histomorphometry

The bone specimens were dehydrated in graded concentrations of ethanol and embedded without decalcification in the modified methyl methacrylate (MMA) using our previously established protocol^27^,^30^. Frontal sections for trabecular bone were obtained from the proximal tibia at a thickness of 15 mm with Leica SM2500E microtome (Leica Microsystems, Germany). Trabecular sections from the proximal tibia were either performed modified Goldner’s trichrome staining for analysis of static parameters or left unstained for collection of fluorochrome-based data. Bone static histomorphometric analysis for osteoblasts surface (Ob.S/BS), bone dynamic histomorphometric analyses for mineral apposition rate (MAR) were calculated using professional image analysis software (Image J, NIH, USA) under fluorescence microscope (Leica image analysis system, Q500MC).

### Immunohistochemistry

The right femur was fixed with 4% buffered formalin and embedded with O.C.T. after decalcification with 10% EDTA. The frozen frontal sections (5 um thickness) was cut in a freezing cryostat at −20 °C. The sections were air dried at room temperature, fixed in ice-cold acetone for 10 min, permeablilized with 0.1% Triton X-100 at room temperature for 20 min, and blocked in 5% donkey serum in PBS. The sections were then incubated overnight at 4 °C with antibody to OCN (1:50 dilution; Santa Cruz Biotechnology, USA), CKIP-1 (1: 500; Santa Cruz Biotechnology, USA), and phosphorylated Smad1/5 (1:1000; Cell Signaling Technology, Inc., USA), respectively. Following three washes in PBS, the sections were incubated with Alexa Fluor 555-conjugated secondary antibody (1:300 dilution; Invitrogen) for 1 h. Negative control experiments were done by omitting the primary antibodies. The sections were mounted with the medium containing DAPI (Vector Laboratories). The sections were examined under a fluorescence microscope (Q500MC, Leica image analysis system). H&E staining was performed on the same sections after immunofluorescence staining for confirmation of the bone formation surface. For quantitative analysis, the number of CKIP-1 and OCN co-positive (CKIP-1^+^ & OCN^+^) cells, pSmad1/5 and OCN co-positive (p-Smad1/5^+^ & OCN^+^) cells and OCN^+^ cells at one sections was calculated blindly by two researchers in this study and the average value was used^27^,^30^.

### Statistical analysis

All numerical data was expressed as mean ± standard deviation. For longitudinal repeated measurement data, repeated measures analysis of variance^31^ was conducted to determine if (a) there was a “time effect” and (b) if there was a “time by group interaction effect”. The “time effect” indicates if there was a change over time for the examined variable, and the “time by group interaction effect” indicates if there were different change patterns over time among the examined groups. If *F* values for a given variable were found to be significant during ‘Tests of Within-Subjects Effects’ and/or ‘Tests of Between-Subjects Effects’, each variable measured at a certain time point was compared with that at the time point of siRNA treatment start in each group using repeated-measures ANOVA after re-organizing output by group. On the other hand, inter-group comparison for each variable measured at a certain time point was also performed by multivariate analysis. If *t* values were found to be significant, LSD’s *post hoc* test was used. For the non-repeated measurement data, one-way ANOVA with LSD’s *post hoc* test was performed to determine inter-group differences in the study variables. A statistical software program (SPSS version 19.0, IBM SPSS Statistics, USA) was used and *P* < 0.05 was considered to be statistically significant.

## Additional Information

**How to cite this article**: Liu, J. *et al*. Targeting osteoblastic casein kinase-2 interacting protein-1 to enhance Smad-dependent BMP signaling and reverse bone formation reduction in glucocorticoid-induced osteoporosis. *Sci. Rep.* **7**, 41295; doi: 10.1038/srep41295 (2017).

**Publisher's note:** Springer Nature remains neutral with regard to jurisdictional claims in published maps and institutional affiliations.

## Supplementary Material

Supplementary Files
