# Supplementary material for: Targeting osteoblastic casein kinase-2 interacting protein-1 to enhance Smad-dependent BMP signaling and reverse bone formation reduction in glucocorticoid-induced osteoporosis
Source: Sci Rep. 2017 Jan 27;7:41295. doi: 10.1038/srep41295 (PMC5269586; doi:10.1038/srep41295)
Supplement: Supplementary Files [file srep41295-s1.pdf]

Targeting osteoblastic casein kinase-2 interacting protein-1 to enhance Smad-dependent BMP signaling and reverse bone formation reduction in glucocorticoid-induced osteoporosis

Jin Liu[1, 3-6 #], Changwei Lu[1,2,7 #], Xiaohao Wu[1, 3-6 #], Zongkang Zhang[8], Jie Li[8], Baosheng Guo[1, 3-5], Defang Li[1, 3-5], Chao Liang[1, 3-5], Lei Dang[1, 3-5], Xiaohua Pan[9], Songlin Peng[10], Aiping Lu[1, 3-6 *], Baoting Zhang[8 *], Ge Zhang[1, 3-5 *]

1 Institute for Advancing Translational Medicine in Bone & Joint Diseases, School of Chinese Medicine, Hong Kong Baptist University, Hong Kong SAR, China

2 Department of Orthopaedics, Xi'an Third Hospital, Xi'an, China

3 Institute of Integrated Bioinfomedicine & Translational Science, School of Chinese Medicine, Hong Kong Baptist University, Hong Kong SAR, China

4 Institute of Precision Medicine and Innovative Drug Discovery, School of Chinese Medicine, Hong Kong Baptist University, Hong Kong SAR, China

5 Shenzhen Lab of Combinatorial Compounds and Targeted Drug Delivery, HKBU Institute of Research and Continuing Education, Shenzhen, China

6 School of Basic Medical Sciences, Shanghai University of Traditional Chinese Medicine, Shanghai, China

7 Department of Orthopaedics, Xijing Hospital, The Fourth Military Medical University, Xi’an, China

8 School of Chinese Medicine, Faculty of Medicine, The Chinese University of Hong Kong, Hong Kong SAR, China

9 Department of Orthopaedics and Traumatology, Bao’an Hospital Affiliated to Southern Medical University & Shenzhen 8th People Hospital, Shenzhen, China.

10 Department of Spine Surgery, Shenzhen People’s Hospital, Ji Nan University Second College of Medicine, Shenzhen, China.

* Corresponding Author: Dr. Ge Zhang, zhangge@hkbu.edu.hk, Dr. Aiping Lu, aipinglu@hkbu.edu.hk, Dr. Bao-Ting Zhang zhangbaoting@cuhk.edu.hk

[#] Equal contribution

Supplementary Table 1 Demography of Patients Included in This Study

GIO Group* (n=10)			Control Group** (n=10)		
Gender	Age	Site#	Gender	Age	Site#
F	45	Distal radius	M	48	Distal radius
M	53	Femur shaft	F	52	Femur shaft
M	47	Femur shaft	F	45	Femur shaft
F	60	Lumbar	M	59	Lumbar
M	59	Lumbar	F	63	Lumbar
M	48	Proximal femurs	M	45	Proximal femurs
F	57	Proximal femurs	F	55	Proximal femurs
M	43	Proximal femurs	M	42	Proximal femurs
F	45	Proximal femurs	F	47	Proximal femurs
F	59	Proximal femurs	M	61	Proximal femurs

*: The patients in GIO group had confirmed history of continuous usage of oral glucocorticoid for more than 6 months; their BMD had also been historically assessed as serious osteoporosis before facture by dual energy X-ray absorptiometry in out-patient clinic. **: The patients in control group were age-matched and had alike fractures, but they did not suffer from serious systemic disease in the previous 5 years and had no history of using glucocorticoid. #: The injury mechanism was falling from a standing height without obvious violence. M, male; F, female; Site, fracture site.

Supplementary Table 2. Body weight of mice treated with or without prednisolone for 4 weeks.

Body weight (gm)	**Baseline (n=8)**	**Prednisolone (n=8)**	**Control (n=8)**
	40.49±6.49	38.66±7.59	40.51±3.46
	**CKO-Baseline (n=8)**	**CKO- Prednisolone (n=8)**	**CKO-VH (n=8)**
	43.71±5.97	42.19±6.02	42.73±6.65
	**CKO Control-Baseline (n=8)**	**CKO Control-Prednisolone (n=8)**	**CKO Control-VH (n=8)**
	40.71±4.35	39.73±4.23	39.25±6.65
	**Smad1-Baseline (n=8)**	**Smad1- Prednisolone (n=8)**	**Smad1-VH (n=8)**
	42.83±5.09	42.01±4.92	43.12±6.08
	**Smad1 Control-Baseline (n=8)**	**Smad1 Control- Prednisolone (n=8)**	**Smad1 Control-VH (n=8)**
	39.74±3.65	38.14±5.83	40.32±3.55

Note: All data were mean ± s.d. Baseline: Baseline of before glucocorticoid treatment. Prednisolone: prednisolone treatment; VH: Vehicle treatment. CKO: osteoblast-specific Ckip-1-knockout mice. Smad1: osteoblast-specific Smad-1-overexpressed mice. Control: mice that treated with vehicle.

Supplementary Table 3: Micro-CT data at baseline

	CKO-control	CKO	Smad1-control	Smad1
**BMD (mg/cm3)**	335.75±12.33	371.77±12.85**	336.72±21.58	377.33±23.62*
**BV/TV (%)**	21.89±4.18	28.16±4.75*	23.20±4.23	26.59±4.69
**Tb.Th (μm)**	74.39±5.01	91.24±11.34*	72.07±11.33	78.37±10.35
**Tb.N (1/mm3)**	5.84±0.47	6.65±0.78*	6.03±0.53	6.43±0.57

Supplementary Table 3: The values of micro-CT parameters (BMD, BV/TV, Tb.Th, Tb.N) in each group at baseline. **Note:** All data were mean ± s.d. ** p<0.001 for CKO group *vs.* CKO-control group. * p<0.05 for CKO group *vs.* CKO-control group and Samd1 group *vs.* Smad1-control group. CKO: osteoblasts-specific Ckip-1 knockout mice; Smad1: osteoblasts-specific Smad1 overexpressing mice.

Supplementary Figure Legend

Supplementary Figure 1 Highly expressed CKIP-1 together with downregulated Smad-dependent BMP signaling and decreased bone formation in GIO. Full-length electrophoretic bands on the intra-osseous protein expression of CKIP-1, pSmad1/5, Smad1/5, Smurf1 and MEKK2 in GIO and Control groups.

Supplementary Figure 2 Decreased bone formation in GIO mice. (a) The values of micro-CT parameters (Tb.Th and Tb.N) in each group. **(b)** The values of bone histomorphometric parameter (Ob.S/BS) in each group. **(c)** Representative micrographs of newly mineralized bone assessed by xylenol (red) and calcein (green) labeling (upper panel) and representative images of Goldner's trichrome staining at the left proximal tibiae in each group. Scale bar: 50um. **(d)** Bone histomorphometric parameters (N.Oc/ BS and Oc.S/ BS) at proximal tibia from each group. **(e)** Representative micrographs illustrating the procedures of laser captured microdissection. Left: the fluorescence micrograph of OCN+ cell staining (green) merged with DAPI counter-staining (blue), Middle: the light micrograph of the adjacent cryosection of proximal tibia before LCM. Right, the light micrograph of the adjacent cryosection of proximal tibia after LCM. Red line areas in the middle and right images highlighted the LCM area.

Supplementary Figure 3 The effects of CKIP-1 knockdown and Smad1 overexpression on CKIP-1 and Smad1/5 protein expression in glucocorticoid-treated MC3T3-E1 cells *in vitro*. (a) Schematic diagram of the experimental design to examine the effect of glucocorticoid (GC) treatment on the osteogenic activity of MC3T3-E1 cells *in vitro*. **(b)** Quantification of CKIP-1 and Smad1/5 protein levels in GT and Control groups at day 3, 5 and 7, respectively. **(c)** Representative electrophoretic bands on the ubiquitination of total Smad1 in GT and Control groups at day 3, 5 and 7, respectively. Polyubiquitinated Smad1 was detected by anti-ubiquitin immunoblot analysis after precipitation of Smad1 in the cell lysis of MC3T3-E1 cells pretreated with proteasome inhibitor MG132. **(d)** Schematic diagram of the experimental design to examine the effect of CKIP-1 knockdown on the osteogenic activity of GC-treated MC3T3-E1 cells *in vitro*. **(e)** Quantification of CKIP-1 and Smad1/5 protein levels in RNAi, VC and NC groups at day 3, 5 and 7, respectively. **(f)** Representative electrophoretic bands on the ubiquitination of total Smad1 in RNAi, VC and NC groups at day 3, 5 and 7, respectively. Polyubiquitinated Smad1 was detected by anti-ubiquitin immunoblot analysis after precipitation of Smad1 in the cell lysis of MC3T3-E1 cells pretreated with proteasome inhibitor MG132. **(g)** Schematic diagram of the experimental design to examine the effect of Smad1 overexpression on the osteogenic activity of GC-treated MC3T3-E1 cells *in vitro*. **(h)** Quantification of CKIP-1 and Smad1/5 protein levels in Smad1 and Vector groups at day 3, 5 and 7, respectively. **Note:** All data were mean ± s.d. * p<0.05 for GT group *vs.* Control group, RNAi group *vs*. NC and VC group or Smad1 group *vs.* Vector group in the corresponding study.

Supplementary Figure 4 Characterization of osteoblast-specific *Ckip-1* knockout mice. (a-c) Schematic diagram for development strategy to generate osteoblast-specific *Ckip-1* knockout (cKO) mice. **(d)** The *Ckip-1* mRNA levels in bone versus non-bone tissues (left) and osteoblasts (OBs) versus non-osteoblasts (Non-OBs) (left) from cKO mice and wildtype (WT) littermates. **(e)** Representative images of Goldner's trichrome staining at the left proximal tibiae in cKO and WT mice with glucocorticoid treatment. Scale bar: 50um. **(f)** Bone histomorphometric parameters (N.Oc/ BS and Oc.S/ BS) at proximal tibia from each group. Base: Baseline of before glucocorticoid treatment. PNL: prednisolone treatment; VH: Vehicle treatment.

Supplementary Figure 5 Characterization of osteoblast-specific *Smad1* knock-in mice. (a-c) Schematic diagram for

development strategy to generate osteoblast-specific *Smad1* knock-in (*Osx/Smad1*) mice. **(d)** The *Smad1* mRNA levels in bone versus non-bone tissues (left) and osteoblasts (OBs) versus non-osteoblasts (Non-OBs) (left) from *Osx/Smad1* mice and control mice. **(e)** Representative images of Goldner's trichrome staining at the left proximal tibiae in *Osx/Smad1* and control mice with glucocorticoid treatment. **(f)** Bone histomorphometric parameters (N.Oc/ BS and Oc.S/ BS) at proximal tibia from each group. Scale bar: 50um. Base: Baseline of before glucocorticoid treatment. PNL: prednisolone treatment; VH: Vehicle treatment.

Supplementary Figure 6 The effect of therapeutic inhibition of CKIP-1 within osteoblast on bone formation in glucocorticoid-treated mice. Representative images of Goldner's trichrome staining at the left proximal tibiae in each group. Baseline: mice sacrificed before glucocorticoid treatment, GIO: mice administrated with phosphate buffer solution (negative control), GIO+Veh: mice administrated with delivery system only, GIO+NC: mice administrated with nonsense RNA negative control with delivery system, GIO+siRNA: mice administrated with CKIP-1 siRNA encapsulated within osteoblast-targeting delivery system, Control: mice that were not treated with glucocorticoid.

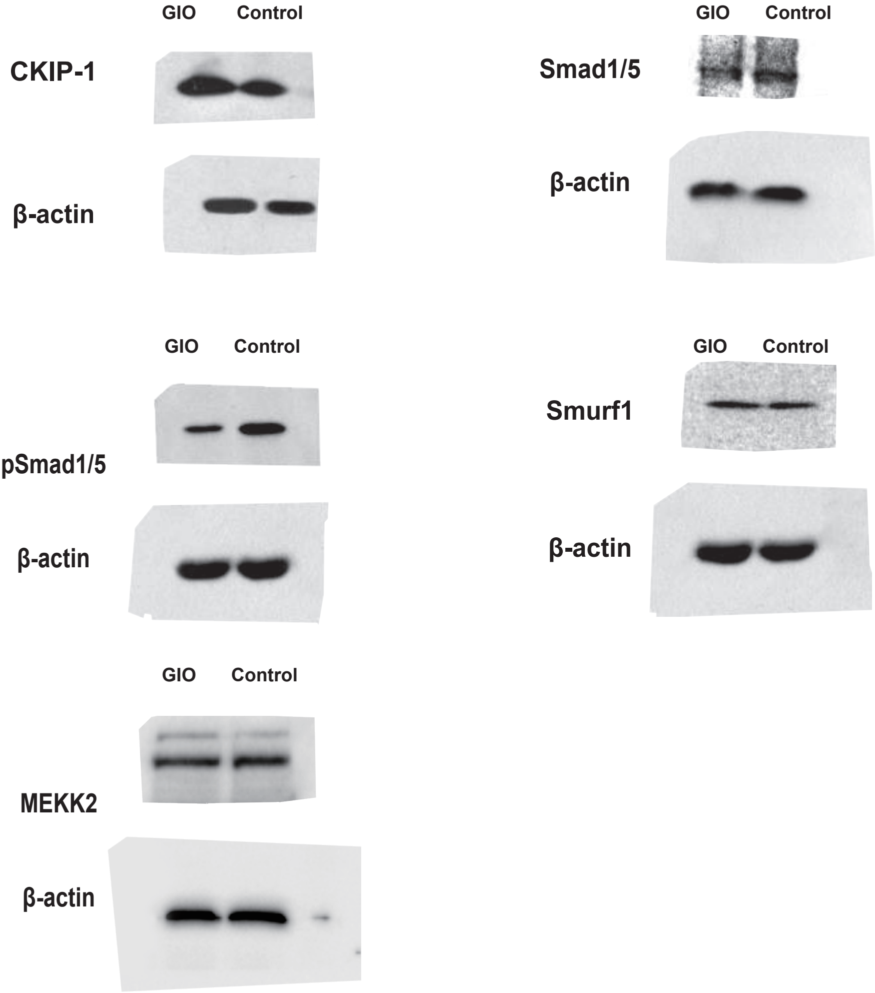

Supplementary Figure 1

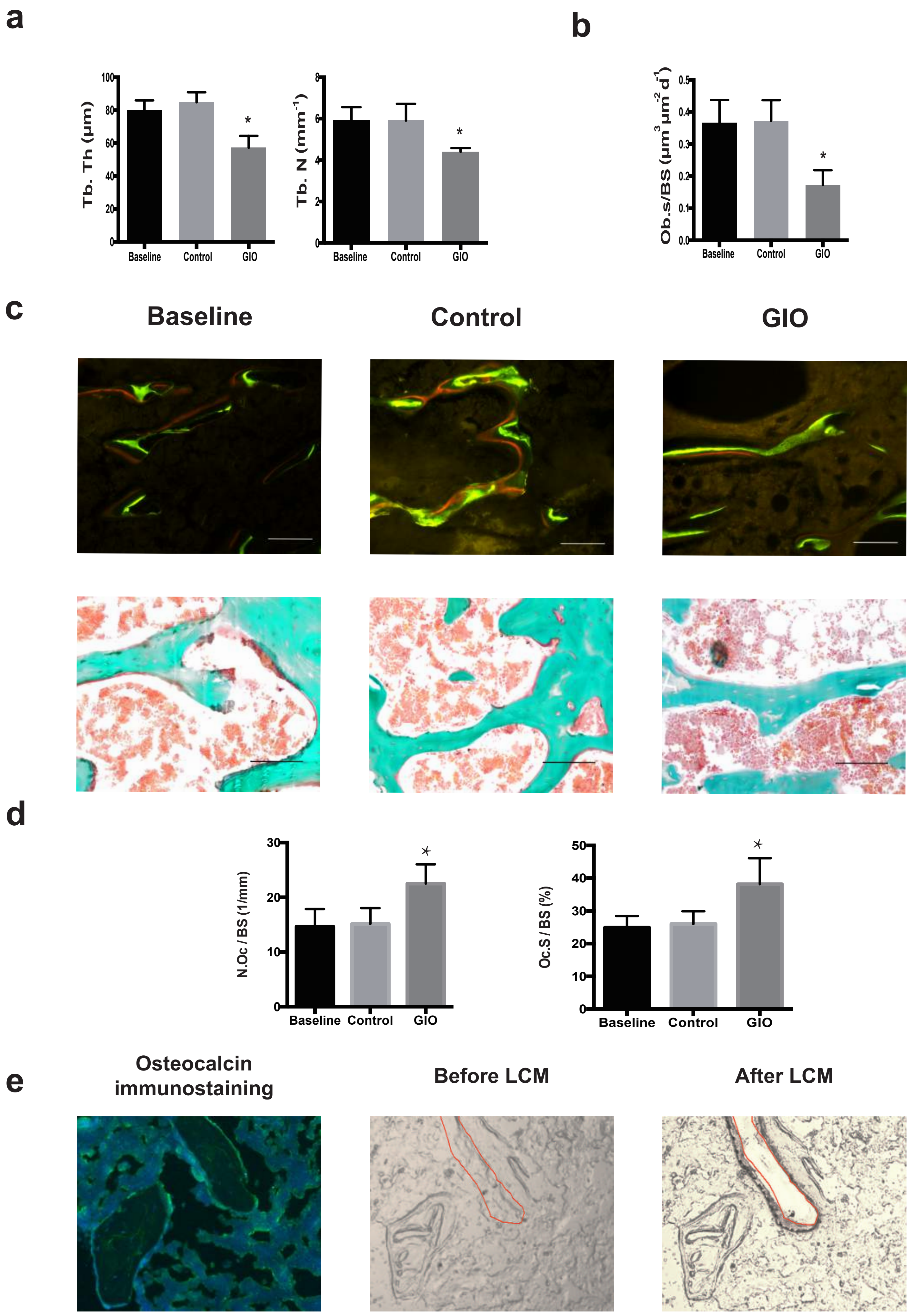

Supplementary Figure 2

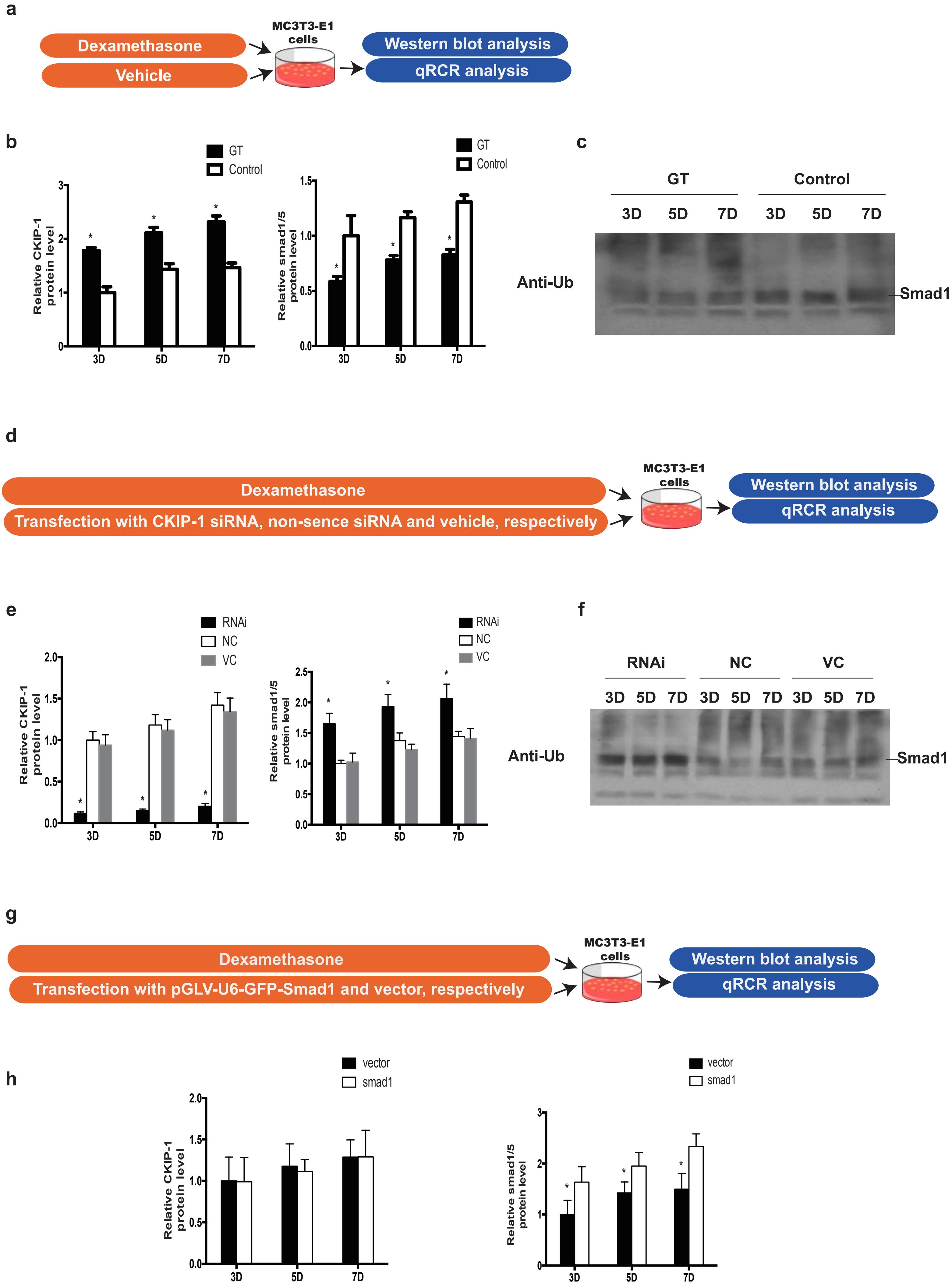

Supplementary Figure 3

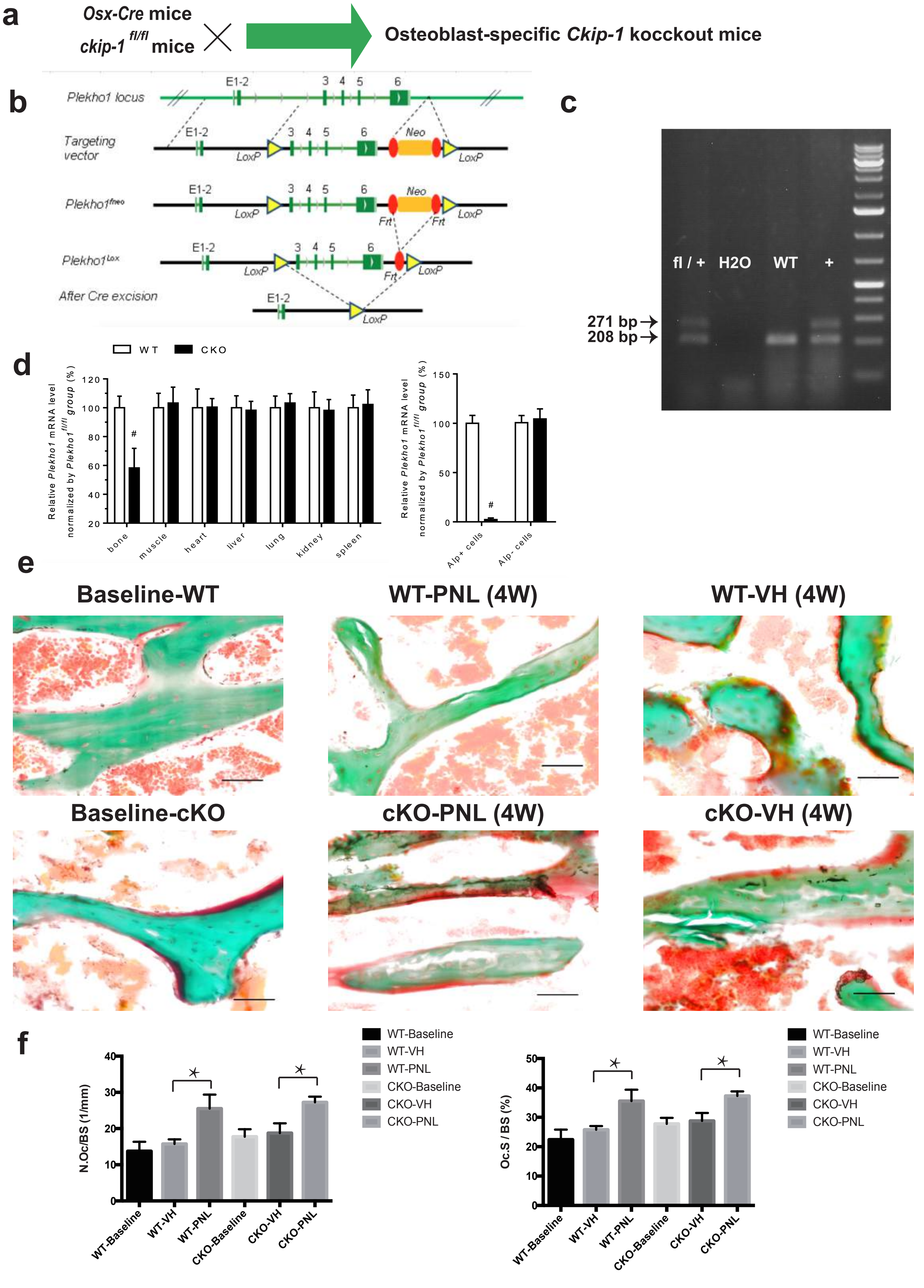

Supplementary Figure 4

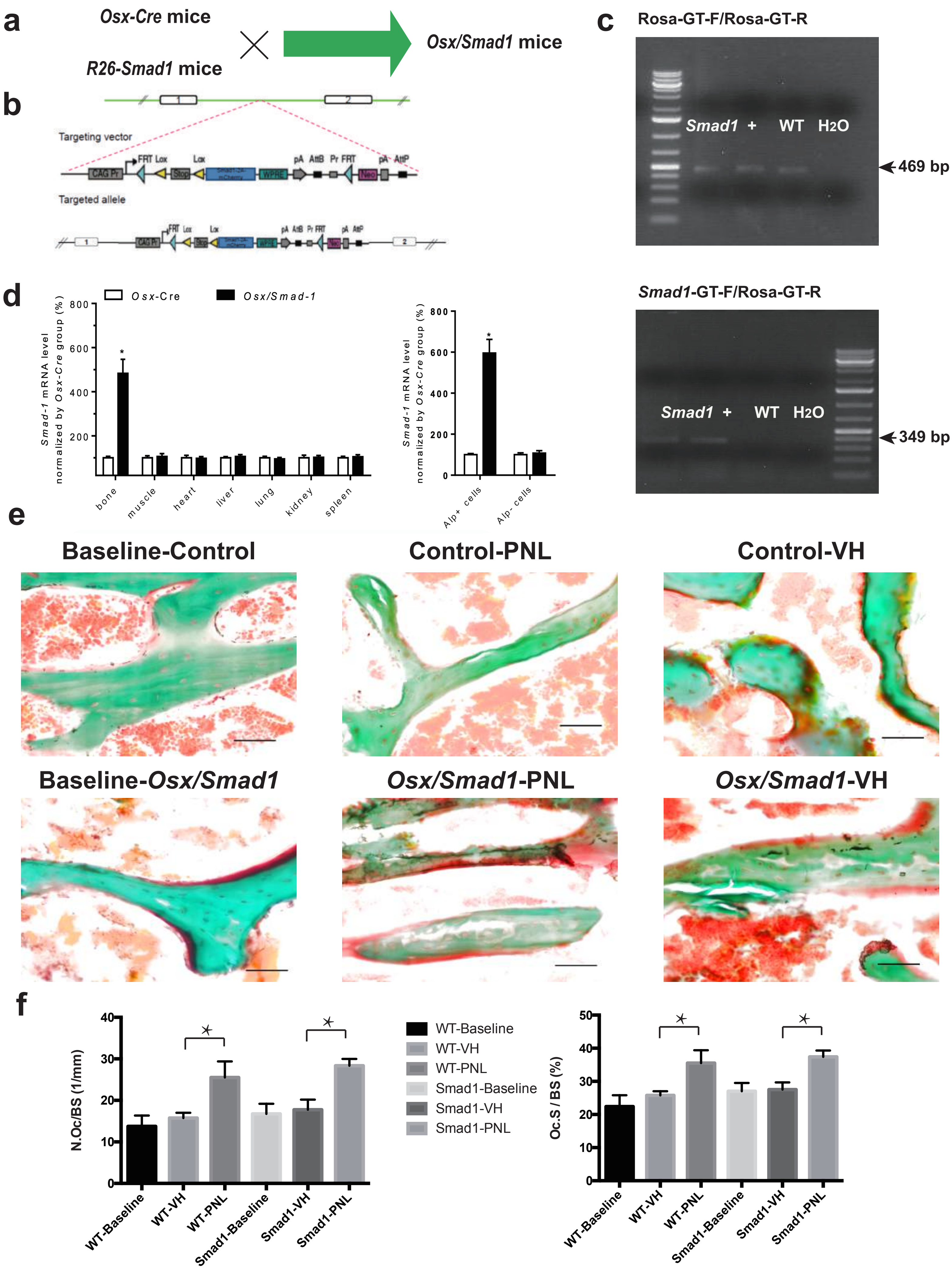

Supplementary Figure 5

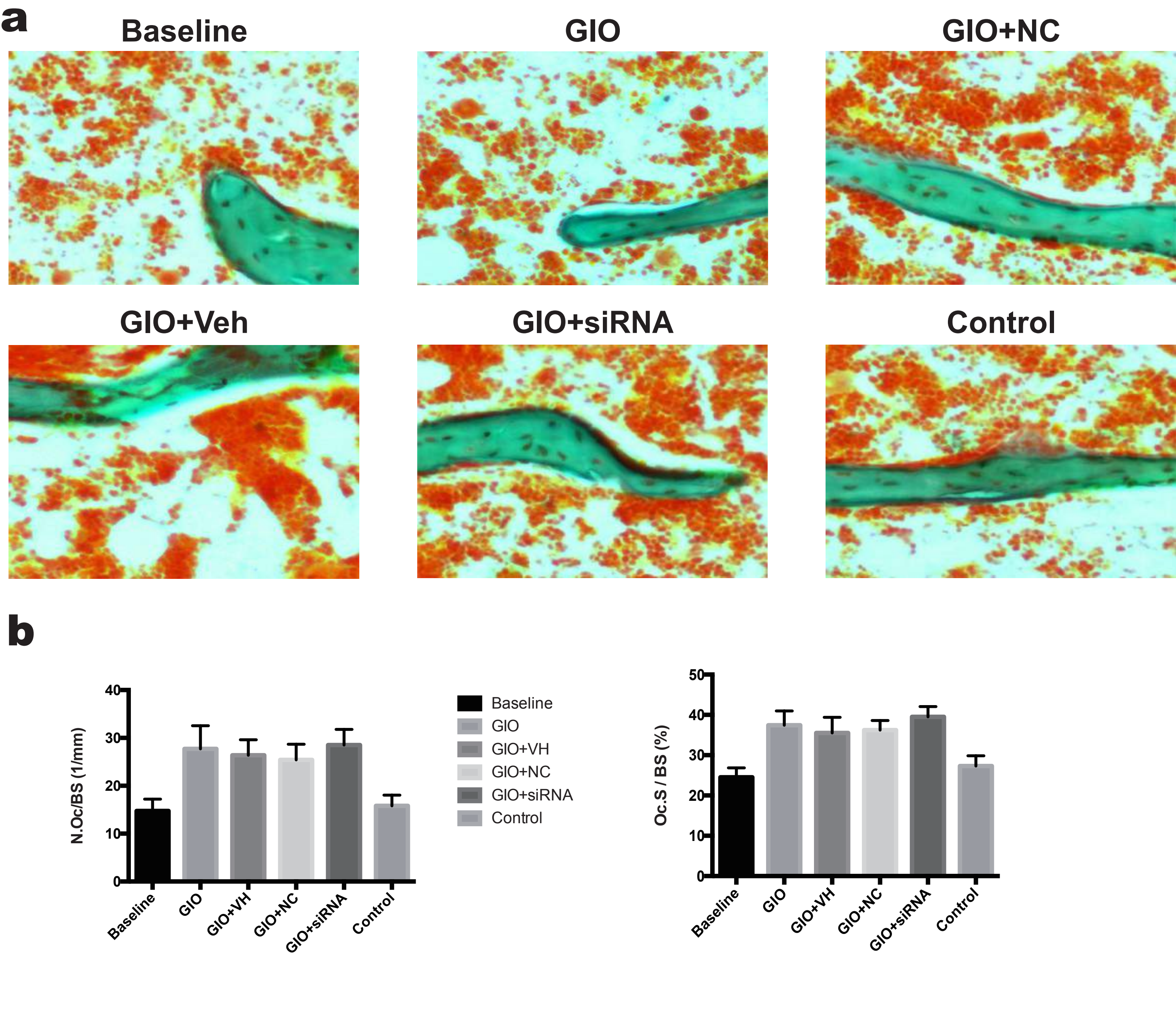

Supplementary Figure 6
